# Hypertension and arterial stiffness in heart transplantation patients

**DOI:** 10.6061/clinics/2016(09)02

**Published:** 2016-09

**Authors:** João David de Souza-Neto, Ítalo Martins de Oliveira, Hermano Alexandre Lima-Rocha, José Wellington Oliveira-Lima, Fernando Bacal

**Affiliations:** I Hospital de Messejana Dr. Carlos Alberto Studart Gomes, Unidade de Transplante e Insuficiência Cardíaca, Fortaleza/CE, Brazil; II Universidade Federal do Ceará, Departamento de Saúde Comunitária, Fortaleza/CE, Brazil; III Universidade Estadual do Ceará, Departamento de Saúde Comunitária, Fortaleza/CE, Brazil; IV Universidade de São Paulo USP, Departamento de Cardiologia, São Paulo/SP, Brazil

**Keywords:** Vascular Stiffness, Hypertension, Graft Rejection, Blood Pressure Monitoring Ambulatory

## Abstract

**OBJECTIVES:**

Post-transplantation hypertension is prevalent and is associated with increased cardiovascular morbidity and subsequent graft dysfunction. The present study aimed to identify the factors associated with arterial stiffness as measured by the ambulatory arterial stiffness index.

**METHODS:**

The current study used a prospective, observational, analytical design to evaluate a group of adult heart transplantation patients. Arterial stiffness was obtained by monitoring ambulatory blood pressure and using the ambulatory arterial stiffness index as the surrogate outcome. Multivariate logistic regression analyses were performed to control confounding.

**RESULTS:**

In a group of 85 adult heart transplantation patients, hypertension was independently associated with arterial stiffness (OR 4.98, CI 95% 1.06-23.4) as well as systolic and diastolic blood pressure averages and nighttime descent.

**CONCLUSIONS:**

Measurement of ambulatory arterial stiffness index is a new, non-invasive method that is easy to perform, may contribute to better defining arterial stiffness prognosis and is associated with hypertension.

## INTRODUCTION

Globally, heart transplantation is indicated for the treatment of heart failure refractory to clinical, surgical and interventional treatments. Transplantation survival has improved significantly in recent decades, mainly due to the emergence of new immunosuppressants 1, which have also promoted greater graft survival 2.

However, post-transplantation hypertension is a major cardiovascular problem. The vasoconstrictor effect of immunosuppressants, including cyclosporin, plays an important role in generating this hypertension 1-3. Additionally, denervation and the consequent increase in heart rate have important implications for exercise, arterial stiffness, graft vascular disease (GVD), acute rejection and other clinical parameters 4,5. Furthermore, patients undergoing heart transplantation present loss of modulation of blood pressure and heart rate during sleep 6.

Data from the International Society for Heart and Lung Transplantation (ISHLT) show that hypertension is present in 50–90% of transplantation patients and is associated with increased cardiovascular morbidity and mortality 7. The Guidelines for the Care of Heart Transplant Recipients specify the use of ambulatory blood pressure monitoring (ABPM) as a class IIa recommendation and level C evidence for assessing hypertension in patients referred for heart transplantation 8,9. One consequence of hypertension is increased arterial stiffness, which leads to cardiovascular events, GVD and increased mortality in transplantation patients 10.

Pulse wave velocity (PWV) and the augmentation index (AIx) are the standard methods of assessing arterial stiffness. However, the ambulatory arterial stiffness index (AASI), derived from the ABPM 11, has been proposed as a new indicator for risk and outcome prediction 12. Recent studies have shown that the AASI can predict cardiovascular morbidity and mortality and cerebrovascular accidents (CVA) 12 and is also a strong predictor of incomplete block of right branch (IBRB) in normotensive patients 12.

GVD remains the main late complication after heart transplantation and is responsible for many deaths after the first year post-transplantation. Furthermore, its association with hypertension and arterial stiffness is well demonstrated 13,14. Therefore, identifying risk factors, including hypertension, for arterial stiffness in transplantation patients, especially using a low-cost, no-risk method, may have clinical importance through identifying the best candidates for assessment. There is a paucity of literature concerning the assessment of arterial stiffness in the transplantation population, especially with respect to heart transplantation.

This work aims to identify the factors associated with arterial stiffness as measured by the AASI, thereby identifying the prognostic utility of this methodology.

## PATIENTS AND METHODS

### Study outline

This study used a prospective, observational, analytical design to measure arterial stiffness using a surrogate outcome index, AASI, as measured by ABPM in heart transplantation patients.

### Study location

The present study was conducted at the Messejana Heart and Lung Hospital, Ceará, Northeast Brazil. Data were collected from March 2012 to June 2013. The patients included in the study were followed until December 2014.

### Population and sample

The study participants were adult patients who had undergone heart transplantation before June 2013. Although there is information available in the literature on arterial stiffness in hypertensive patients, most studies 15-17 have focused on arterial stiffness in combination with other cardiovascular diseases 15 and in renal transplantation recipients and diabetic patients 18-20. In the current study, we hypothesized that arterial stiffness would be present in patients with potential predisposing factors such as hypertension and chronological age. We recruited heart transplantation patients who met the following inclusion criteria: adults (over 18 years of age) who underwent transplantation more than 6 months before enrollment. Patients who did not agree to provide voluntary, informed consent were excluded from participation. All adult patients identified during the study period were invited to participate in the study, and a total of 97 patients were enrolled. Twelve patients were excluded for reasons such as not providing consent, limited mobility, living in another state and refusing to adhere to the planned evaluations.

### Variables

Clinical and laboratory data were stored in a special clinical record and transported to a specific database. All patients underwent clinical examination from March 2012 to June 2013 before being included in the study protocol. The examination included a routine laboratory evaluation, a clinical protocol with biochemical and hematological examination and an evaluation of the dosage of immunosuppressants (cyclosporin serum, tacrolimus, sirolimus and everolimus) obtained at time “0” from use.

Transthoracic echocardiography was performed for all transplantation patients to evaluate graft function. Endomyocardial biopsy was performed at 1, 2, 3 and 6 months post-transplantation, when the patient completed their first year of transplantation, or at any time there was suspicion of graft rejection. All analyses were conducted by the same pathologist in the study institution following the ISHLT classification.

Coronary angiography was performed on completion of the first year after transplantation or at any time suspicion of GVD arose. If more than 6 months elapsed since the participant’s last coronary angiography, the procedure was repeated. GVD was indicated by coronary angiography showing at least a 50% lesion in a coronary artery. All procedures were conducted at the study institution.

ABPM was performed for all patients for a 24-hour period. The monitor used was a Meditech® ABPM 04 (Meditech Ltd, Hungary), with a program specific for the calculation of AASI. The measurement protocol followed the American Hypertension Guidelines with 15/15-minute measurements during the monitoring period and 30/30-minute measurements during sleep, with monitoring starting at 7:00 hours and ending at 22:59 hours. Abnormality criteria were mapped according to the guidelines. Analysis of ABPM and AASI data was performed by a single doctor, who was blinded to patient identification.

### Statistical analysis

Distributions of continuous variables were described using mean values and standard deviations. Categorical variables were described by relative frequency categories. The normality of all numeric variables was tested using the Kolmogorov-Smirnov test. The variables that showed an abnormal distribution were transformed with Tukey’s ladder and the best possible transformation was chosen.

An interaction test was used to identify possible multiplicative interactions between transplantation and hypertension in the development of arterial stiffness through a logistic regression model. A bivariate analysis was performed with parametric tests and simple regression models using arterial stiffness as a binary variable. Finally, multiple logistic regression was performed to control confounding between determinants with variables having *p* values up to 0.1 in the bivariate analysis.

### Ethics

The present study obtained approval from the Ethics and Research Committee of the study institution under the number 826/2011 and all enrolled patients provided written informed consent.

## RESULTS

The study group included 85 transplantation patients, 29 of whom presented with arterial stiffness. Univariate analysis was performed to analyze the determinants of arterial stiffness. After analyzing the patients’ comorbidities and the impacts of diabetes, hypertension, peripheral artery disease and coronary heart disease, it was found that all of these factors are significantly related to arterial stiffness (20.8%, 50%, 7.9%, and 46.1% if stiffness was present in a patient *versus* 3.4%, 20.3%, 0, and 16.1% if not). Patient gender and age did not appear to be related to arterial stiffness (Table 1).

The patients’ laboratory evaluations indicated that creatinine levels were related to arterial stiffness (1.64 for patients with arterial stiffness and 1.16 for patients without arterial stiffness, *p*<0.001), as well as levels of urea (*p*=0.006) and of high-density cholesterol (*p*=0.015) (Table 2).

**Figure 1:**
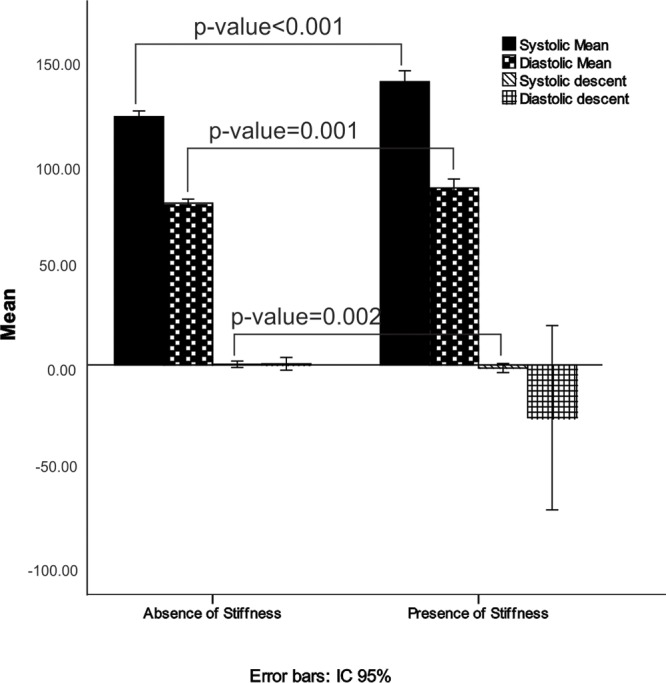
ABPM averages for transplantation patients with and without the presence of arterial stiffness.

Analysis of the ABPM data showed that average systolic and diastolic blood pressure measurements were related to the development of arterial stiffness (the mean systolic blood pressure was 140.58 in the stiffness group and 123.25 in the group without stiffness, *p*<0.001). Systolic descent also showed significance, as shown in Table 3 and Figure 1.

Heart transplantation-specific variables, especially GVD and time from transplantation, were also associated with arterial stiffness (Table 4).

Table 5 contains data regarding the use of immunosuppressive drugs by the patients with and without arterial stiffness as measured by the AASI. Mycophenolate mofetil use was associated with an increase in the occurrence of stiffness (OR 2.91, CI 95% 1.04-8.15).

Finally, the multivariate analysis of the determinants identified arterial stiffness as independently associated with hypertension. Hypertension was over four times more prevalent in transplantation patients with stiffness (OR 4.98, CI 95% 1.06-23.4) and with an absence of diastolic nighttime descent. Additionally, GVD (OR 14.9, CI 95% 2.0-107.5), diabetes (OR 77.8, CI 95% 3.88-1561.4) and elevated creatinine levels (OR 80.7, CI 95% 5.5-176.6) were highly associated with arterial stiffness. All variables identified as significant in the regression are presented in Table 6.

## DISCUSSION

In recent years, arterial stiffness has been shown to be an important marker of cardiovascular events and CVA in the general population, especially in patients with chronic kidney disease or diabetes 15,22. The AASI, a new measure for determining arterial stiffness, was previously used to show that arterial stiffness is associated with injury to target organs and cardiovascular morbidity and mortality 15. However, no studies have investigated using the AASI as a marker of arterial stiffness in heart transplantation patients.

Our transplantation patients presented higher average diastolic blood pressure and smaller nocturnal systolic descents and diastolic levels than the normal population. These factors are probably secondary to cardiac denervation and the use of immunosuppressants and can lead to worsening endothelial function in the transplantation population.

The literature shows that patients who have a normal decrease in blood pressure during sleep have a reduction in cardiac output with maintenance of peripheral resistance, while heart transplantation patients have lower reductions in cardiac output and in peripheral resistance due to a relative absence of nighttime descent 23. However, the literature does not report on what impact the absence of nighttime descent has on the risk of arterial stiffness. Additionally, blood pressure variables obtained through the ABPM (average systolic and diastolic blood pressure, average systolic and diastolic descent) were significant risk factors for increased arterial stiffness, suggesting the importance of hypertension as a determinant of arterial stiffness in transplantation patients 24-26.

In a cohort of 330 renal transplantation patients, Mitchell et al. observed significant increases in PWV (a marker of arterial stiffness) with age, systolic blood pressure and pulse pressure. They concluded that PWV is a strong predictor of all-cause mortality in the renal transplantation population (*p*<0.001) 27.

A number of publications and reviews of various physiopathological conditions have reported associations between age, genetic factors, physical inactivity, smoking 28, obesity, hypercholesterolemia 29, coronary artery disease, metabolic syndrome, heart failure, stroke, chronic kidney disease, rheumatoid arthritis, systemic lupus erythematosus and vasculitis 30 and increased arterial stiffness.

Hypertension is strongly associated with an increased risk of arterial stiffness 17. Other comorbidities also have an important relationship with increased risk, as noted in previous studies 31-33. In our study, comorbidities were also associated with the development of arterial stiffness, such as diabetes mellitus and dyslipidemia. Diabetes was the most important factor associated with increased arterial stiffness. A recent systematic review reported that there is still no consensus on this association; thus, the current work provides important support to strengthen the association 34.

Routine clinical applications for measuring arterial stiffness may be expanded, as many studies have demonstrated the predictive value of arterial stiffness for the risk of cardiovascular events and its mitigation through the use of antihypertensive, antidiabetic and cholesterol-lowering drugs, among others 35-36.

Our multivariate analysis showed that the association between hypertension and arterial stiffness was not confounded by other factors. Additionally, other independent factors were identified, including diabetes mellitus, GVD and elevation of creatinine levels 37,38.

The loss of renal function, measured as increased creatinine and urea levels, was the most important laboratory factor identified for increased risk of arterial stiffness in transplantation patients. This finding is consistent with the literature 39-41. In another study, arterial stiffness was a strong predictor of cardiovascular events in renal transplantation recipients 42.

Multiple factors are involved in generating hypertension in heart transplantation patients; these include immunosuppressive therapy, increased sensitivity of beta-adrenergic receptors due to donor heart denervation, worsening of endothelial function and drug nephrotoxicity 43,44.

GVD is the main complication of heart transplantation. Identified risk factors for GVD include hypertension, obesity, diabetes, and smoking. In our study, GVD was related to arterial stiffness, and its determinants were also associated 45.

Arterial stiffness is an independent predictor of cardiovascular events and all-cause mortality. The main risk factors for arterial stiffness include hypertension, diabetes, dyslipidemia, and chronic kidney disease. Heart transplantation patients who have these risk factors should be screened for early development of arterial stiffness.

The data in the current study highlight the need for early detection of hypertension and routine indication of ABPM in heart transplantation patients with risk factors. Based on our results, arterial stiffness should be analyzed using the AASI or a similar evaluation method with the goal of early intervention to reduce adverse events.

The present study was limited by the small number of transplantation patients included. However, this is a unique population, which by nature is difficult to select. Another limitation was the lack of comparison between AASI arterial stiffness measurements and another group measured using standard methods such as the PWV or AIx, which may have improved the accuracy of the analysis. However, for heart transplantation patients as a specific group, our study reached its goals of identifying a method of analysis for arterial stiffness that is non-invasive, simple to perform and easily adaptable for future studies.

This study concluded that hypertension was independently associated with estimated arterial stiffness in a group of adult heart transplantation patients. Renal dysfunction, diabetes and absence of diastolic descent were identified as determinants of arterial stiffness. Measuring the AASI offers a new, non-invasive method that is easy to perform and is indicative of hypertension. This method may assist in defining therapeutic strategies for transplantation patients and could contribute to improving prognosis.

## AUTHOR CONTRIBUTIONS

Souza-Neto JD, Oliveira IM, Bacal F, Lima-Rocha HA and Oliveira-Lima JW provided substantial contributions to the conception and design of the study and to critically revising the manuscript for important intellectual content. Souza-Neto, Oliveira IM and Lima-Rocha HA were responsible for the analysis and interpretation of data and for drafting the manuscript. All of the authors read and approved the final version of the manuscript.

## Figures and Tables

**Table 1:** Univariate analysis results showing the relationships between sociodemographic variables, cardiomyopathies, comorbidities and arterial stiffness index (n = 85)^1^.

	Arterial stiffness - AASI				
	Yes	No	OR	CI 95%	*p*-value
Age (years)	58.4 (±8.3)	54.2 (±11.4)	0.95	0.91-1.00	0.061
Male	21 (88.3)	49 (80.2)	1.71	0.44-6.71	0.439
Myocardiopathy					0.092
Idiopathic	7 (29.3)	23 (38.1)	0.55	0.56-5.35	0.605
Chagasic	2 (8.2)	14 (23.3)	1.16	0.08-15.4	0.907
Ischemic	11 (45.8)	9 (15.2)	0.13	0.01-1.35	0.089
Valve	1 (4.2)	5 (8.2)	0.83	0.04-16.9	0.906
Alcoholic	2 (8.3)	4 (6.6)	0.33	0.02-5.02	0.427
Others	1 (4.1)	6 (10.2)	1	-	-
Prior diabetes	5 (20.8)	2 (3.4)	7.76	1.39-43.3	**0.019**
Prior hypertension	12 (50.0)	12 (20.3)	4.08	1.47-11.3	**0.007**
Prior CAD	11 (46.1)	10 (16.1)	4.31	1.51-12.3	**0.006**
Prior PAD	2 (7.9)	0 (0.0)	-	-	-
Prior PLD	8 (33.1)	5 (8.2)	5.60	1.61-19.5	**0.007**

1 The results are expressed in absolute and relative frequencies (%) for categorical variables and in means and standard deviations (± SD) for numeric variables. CAD, coronary arterial disease; PAD, peripheral arterial disease; PLD, dyslipidemia.

**Table 2:** Univariate analysis results showing the relationships between laboratory profiles and arterial stiffness index (n = 85)^1^.

	Arterial stiffness – AASI				
	Yes	No	OR	CI 95%	*p*-value
Fasting blood glucose mg/dL	111.42 (±33.6)	100.0 (±21)	1.02	0.99-1.03	0.086
Glycated hemoglobin	6.19 (±1.27)	6.66 (±7.85)	0.98	0.88-1.09	0.716
Creatinine mg/dL	1.64 (±0.49)	1.16 (±0.26)	56.7	7.4-43.4	**<0.001**
Urea mg/dL	60.29 (±20.14)	47.64 (±15.94)	1.04	1.01-1.07	**0.006**
Total cholesterol mg/dL	182.75 (±57.48)	167.34 (±41.51)	1.01	0.99-1.02	0.174
LDL cholesterol mg/dL	93.34 (±28.97)	105.50 (±45.07	1.01	0.99-1.03	**0.154**
HDL cholesterol mg/dL	37.08 (±8.84)	44.13 (±12.05)	0.94	0.89-0.99	**0.015**
Triglycerides mg/dL	177.54 (±81.68)	141.49 (±73.64)	1.01	1.00-1.01	0.058

1 The results are expressed in absolute and relative frequencies (%) for categorical variables and in means and standard deviations (± SD) for numeric variables. LDL, low-density cholesterol; HDL, high-density cholesterol.

**Table 3:** Univariate analysis results showing the relationships between ABPM data and arterial stiffness index (n = 85)^1^.

	Arterial stiffness – AASI				
	Yes	No	OR	CI 95%	*p*-value
Hypertension	18 (75.2)	13 (21.2)	11.1	3.65-33.5	**<0.001**
Average systolic BP mm/Hg	140.58 (±12.84)	123.25 (±10.97)	1.13	1.07-1.19	**<0.001**
Average diastolic BP mm/Hg	87.83 (±10.41)	80.30 (±7.72)	1.11	1.04-1.18	**0.001**
Systolic descent	-1.54 (±5.36)	0.35 (±6.06)	0.94	0.87-1.03	**0.002**
Diastolic descent	-26.17 (±108.27)	0.56 (±12.37)	0.98	0.95-1.01	0.174
Heart rate	91.41 (±9.19)	94 (±10.52)	1.03	0.98-1.08	0.263

1 The results are expressed in absolute and relative frequencies (%) for categorical variables and in means and standard deviations (± SD) for numeric variables. BP, blood pressure.

**Table 4:** Univariate analysis results showing the relationships between post-transplantation variables and arterial stiffness index (n = 85)^1^.

	Arterial stiffness – AASI				
	Yes	No	OR	CI 95%	*p*-value
Graft vascular disease	8 (32.8)	6 (10.2)	4.42	1.33-14.7	**0.015**
Rejection	2 (18.2)	9 (28.1)	0.98	0.16-5.99	1.000
Ventricular dysfunction	4 (16.8)	9 (15.3)	4.58	0.67-31.2	0.282
Time to transplant	5.81 (±3.12)	4.16 (±3.34)	1.16	1.00-1.33	**0.044**

1 The results are expressed in absolute and relative frequencies (%) for categorical variables and in means and standard deviations (± SD) for numeric variables.

**Table 5:** Description of medications used in accordance with the index of arterial stiffness as measured by the ambulatory arterial stiffness index (n = 85).

	Arterial stiffness – AASI				
	Yes	No	OR	CI 95%	*p*-value
CORTICOID	2 (18.2)	9 (81.8)	0.51	0.10-2.58	0.420
MYCOPHENOLATE MOFETIL	10 (45.5)	12 (22.2)	2.91	1.04-8.15	**0.041**
MMS	12 (19.7)	49 (47.8)	0.26	0.09-0.75	**0.012**
CICLOSPORIN	14 (30.4)	32 (69.6)	1.26	0.4-3.2	0.625
TACROLIMUS	2 (11.8)	15 (88.2)	0.28	0.06-1.36	0.116
SIROLIMUS	5 (35.7)	9 (64.3)	1.49	0.44-5.01	0.519
EVEROLIMUS	4 (44.4)	5 (55.6)	2.16	0.52-8.85	0.285

**Table 6:** Multivariate analysis results showing the relationships between time of transplant data and arterial stiffness as measured by the ambulatory arterial stiffness index (n = 85).

Variable	OR	CI 95%	*p*-value
Median age	7.03	1.37-36.2	**0.020**
Hypertension	4.98	1.06-23.4	**0.042**
Prior diabetes	77.8	3.88-1561.4	**0.004**
Graft vascular disease	14.9	2.0-107.5	**0.007**
Diastolic descent	0.98	0.97-1.00	**0.049**
Creatinine			
First tertile	-	-	**0.003**
Second tertile	12.9	1.09-154.6	**0.043**
Third tertile	80.7	5.54-176.6	**0.001**
